# Modification effects of genetic polymorphisms in *FTO*, *IL-6*, and *HSPD1* on the associations of diabetes with breast cancer risk and survival

**DOI:** 10.1371/journal.pone.0178850

**Published:** 2017-06-07

**Authors:** Rui-Mei Zhu, Wei Lin, Wei Zhang, Jun-Ting Ren, Yi Su, Jian-Rong He, Ying Lin, Feng-Xi Su, Xiao-Ming Xie, Lu-Ying Tang, Ze-Fang Ren

**Affiliations:** 1 Department of Statistics and Epidemiology, Guangzhou Key Laboratory of Environmental Pollution and Health Risk Assessment, School of Public Health, Sun Yat-Sen University, Guangzhou, China; 2 Department of Medicine and Therapeutics, Prince of Wales Hospital, The Chinese University of Hong Kong, Shatin, Hong Kong, China; 3 The Guangzhou Women and Children’s Medical Center, Guangzhou, China; 4 The First Affiliated Hospital, Sun Yat-Sen University, Guangzhou, China; 5 The Second Affiliated Hospital, Sun Yat-Sen University, Guangzhou, China; 6 The Cancer Center, Sun Yat-Sen University, Guangzhou, China; 7 The Third Affiliated Hospital, Sun Yat-Sen University, Guangzhou, China; Hospital Israelita Albert Einstein, BRAZIL

## Abstract

The contribution of diabetes to breast cancer remains uncertain among Chinese females, which may result from different genetic factors. We evaluated the associations of diabetes, combined with the polymorphisms in the genes of fat mass and obesity-associated gene (*FTO*), interleukin 6 (*IL-6)*, and heat shock protein 60 (*HSPD1)*, with breast cancer risk and survival in a Chinese Han population. The information on the history of diabetes was collected from 1551 incident breast cancer cases and 1605 age-frequency matched controls in Guangzhou, China. In total, 1168 cases were followed up. Diabetes was associated with both an increased risk of breast cancer [OR (95%CI): 1.67 (1.11, 2.52)] and a poor overall survival and progression free survival for breast cancer patients [HRs (95%CIs): 2.66 (1.10, 6.44) and 2.46 (1.29, 4.70), respectively]. *IL-6* rs1800796 and *HSPD1* rs2605039 had interactions with diabetes on breast cancer risk. Among women with CC genotype of *IL-6* rs1800796 or GG genotype of *HSPD1* rs2605039, diabetic individuals had a remarkably increased risk of breast cancer compared to non-diabetic women with ORs and 95%CIs of 2.53 (1.45, 4.41) and 6.40 (2.29, 17.87), respectively. GT/TT genotypes of *HSPD1* rs2605039 was also associated with a better progression free survival for breast cancer patients [HR (95%CI): 0.70 (0.49, 0.99)]. Our results suggest that the contribution of diabetes to breast cancer risk might be modified by *IL-6* rs1800796 and *HSPD1* rs2605039. Diabetes and *HSPD1* rs2605039 might also influence breast cancer prognosis.

## Introduction

Diabetes is characterized by sustained hyperglycemia and is associated with increased cancer risk in many settings, possibly through proliferative, anti-apoptotic, and metastatic activity [1]. Therefore, a series of studies have been performed to explore the associations of type 2 diabetes mellitus (T2DM), the common type in later life (~95% of all diabetic cases), with the risk and prognosis of cancers [1]. For breast cancer, the association was mainly observed in European females [2]. In Asia, however, the results were not conclusive with both positive [3–6] and null associations [7–9]. Except for different diets and lifestyles, this inconsistency may be explained by genetic predisposition [10].

Hyperglycemia exerts effects on carcinogenic process by concurring with a chronic inflammation state and an associated oxidative stress condition [11–13]; the former is accompanying with an elevated levels of inflammatory cytokines, such as interleukin-6 (*IL-6*) [14], whereas the latter may damage mitochondrial function that is closely related to heat shock protein 60 (hsp60, encoded by *HSPD1*) [15,16]. In addition, as a shared predictor of obesity and diabetes, the fat mass and obesity-associated gene (*FTO*) has been reported to be involved in above pathways [17,18], which may play possible modified roles in the process from diabetes to breast cancer initiation.

Therefore, in the present study, we selected one potentially functional single nucleotide polymorphism (SNP) from each of the three genes, *FTO* rs3751812, *IL-6* rs1800796, and *HSPD1* rs2605039 as genetic factors. We then explored the associations of pre-existing diabetes and the genetic factors with the risk and prognosis of breast cancer. The modification effects of the genetic factors on the associations were further assessed.

## Materials and methods

### Study population

Female breast cancer patients were newly histologically diagnosed in the First and Second Affiliated Hospitals and the Cancer Center of Sun Yat-Sen University in Guangzhou, China, from October 2008 to March 2012. Cancer-free controls, frequency-matched to the cases for age, were recruited from women who attended a health checkup in the same hospitals during the same period. All participants must have resided in the Guangzhou area for at least 5 years. In total, 1,736 cases and 1,773 controls were recruited. Participants with a diagnosis of other cancers, or those refused to complete questionnaires or donate blood samples, were excluded, leaving 1,551 (89.3%) cases and 1,605 (90.5%) controls for this study. Written informed consent was obtained for the interviews and the specimen collections from all individual participants included in the study. This study was approved by the Ethical Committee of the School of Public Health in Sun Yat-Sen University.

### Baseline data collection

Trained interviewers collected information from cases and controls by a face-to-face interview using the same questionnaire. This questionnaire contains the following information: menstrual and reproductive history, life style, family history of cancer, height and weight, and demographic factors. Information on diabetes mellitus was collected from all subjects by questions “Have you ever been diagnosed diabetes mellitus by a physician (including prediabetes and diabetes requiring or not requiring insulin)?” and “If you had diabetes before, what is the age at first diagnosis?” Women who had been diagnosed of diabetes before the time of interview were counted in the group of patients with diabetes mellitus. Clinical characteristics of breast cancer patients were collected from medical records and pathological reports. The statuses of estrogen receptor (ER), progesterone receptor (PR), and human epidermal growth factor receptor 2 (HER2) for breast cancer tissues were determined by pathologists using immunohistochemistry tests. The definition of statuses of ER, PR, and HER2 were previously described in detail [19]. All information was truncated at the reference date, which was the date of diagnosis for cases and the date of completion of the questionnaire for controls.

### Follow-up of the patients

As previously described [20], all patients were followed up at least every 3 months during the first year, and every 6 months during the second and the third year; thereafter, patients were followed up once every year until death or December 31, 2014. The follow-up data were obtained from 1,168 (75.3%) breast cancer cases by means of letter, phone call and outpatient visits with a median follow-up duration of 47.8 months. The following information was acquired: updated contact information, physical conditions (recurrence, metastasis, death, or newly diagnosed diseases with a certain time), post-diagnosis life style, menstrual and weight changes, treatment information (radiotherapy, chemotherapy, hormonal therapy, and surgical options). The primary endpoint for this study was overall survival (OS), defined as the time from diagnosis until death; the patients still alive have been censored at their latest date of follow-up. The second endpoint was progression free survival (PFS), calculated from diagnosis to the date of progression (recurrence or metastasis) or death; the patients still alive without progression had been censored at the latest date of their follow-up. Patients who died of other causes were also censored. During the follow-up period, 109 (9.3%) and 193 (16.5%) patients died or experienced breast cancer progression, respectively.

### Laboratory protocol

The detail methods were described elsewhere [21]. Briefly, blood samples were donated from cases immediately after admission to the hospitals and from controls after the interview, which were stored at −80°C until they were analyzed. According to the manufacturer’s instructions, genomic DNA was extracted from the buffy coats of the participants using the TIANamp Genomic DNA Kit (TianGen Biotech Co., Ltd., Beijing, China) and genotyped by Sequenom (San Diego, California, USA). The details of the primers are described in S1 Table. Duplicate samples (5% of the total) were included for the evaluation of genotyping quality and the concordance rate was 100%. No deviation from the Hardy-Weinberg equilibrium was observed (*P* = 0.274, 0.204, and 0.854 for rs3751812, rs1800796, and rs2605039, respectively, in the control group).

We selected these three SNPs based on the reasons that they have potential functions or are linked with functional SNPs. *FTO* rs3751812 is located in the intron, but it was found to be consistently associated with obesity and lipid parameters in Asian population [22]. *IL-6* rs1800796 is located in the promoter region and were reported to be associated with serum levels of IL-6 [23]. *HSPD1* rs2605039 is an intron SNP, while it is perfectly linked with rs8539, an exon SNP [24].

### Statistical analysis

To explore the differences in demographic characteristics and common risk factors for breast cancer between the cases and controls, we applied Chi-square test (for categorical variables) or Student’s t-test (for continuous variables). Hardy-Weinberg equilibrium test for *FTO* rs3751812, *IL-6* rs1800796, and *HSPD1* rs2605039 was evaluated by a goodness-of-fit Chi-square test to compare the observed genotype frequency with the expected one among the controls. Further, we used multivariate logistic regression to assess the associations of diabetes, as well as genetic variations, with breast cancer risk, adjusting for potential confounders, which are age (continuous), age at menarche, marital status, education, body mass index (BMI), parity, menopausal status, breastfeeding, physical activity, and family history of breast cancer. The mutant homozygotes and heterozygotes were combined assuming a dominant model for three genetic variations [20]. The interactions between diabetes and genotypes of three SNPs on breast cancer risk were evaluated by both multiplicative and additive models. We tested for multiplicative interaction by including the product term in multivariate logistic regression. Additive interaction was assessed with method proposed by Rothman [25,26]. Survival curves were made by the Kaplan—Meier method and compared by the log-rank test. The Cox proportional hazards model was applied for univariate and multivariate analysis to identify prognostic factors. The multivariate model included clinical characteristics of age at diagnosis (continuous), ER and HER2 statuses, clinical stages, chemotherapy, and surgical options. All statistical tests were 2-tailed with *P* < 0.05 considered to be significant. Statistical analyses were performed using SPSS 20.0 and SAS 9.2.

## Results

Demographic characteristics and breast cancer related factors were shown in Table 1. The distributions of age, marital status, BMI, age at menarche, age at menopause, parity, breastfeeding and family history of breast cancer did not significantly differ between the cases and controls. However, as compared to the controls, breast cancer patients tended to be premenopausal, less educated, and having less physical activity (Table 1).

**Table 1:** Characteristics of breast cancer cases and controls.

Characteristic	Case n (%)	Control n (%)	*P*-value ^a^
**Age** (years)			
≤40	419 (27.0)	444 (27.7)	
41~60	907 (58.5)	931 (58.0)	
≥61	225 (14.5)	230 (14.3)	0.919
Mean (SD)	48.35 (11.38)	48.39 (11.46)	0.933 ^b^
**Education**			
Junior middle school or below	736 (47.5)	596 (37.1)	
Senior middle school	399 (25.7)	581 (36.2)	
College or above	340 (21.9)	385 (24.0)	<0.01
Unknown	76 (4.9)	43 (2.7)	
**Marital status**			
Never married	64 (4.1)	62 (3.9)	
Married/living as married	1350 (87.0)	1386 (86.4)	
Separated/widow	95 (6.1)	104 (6.5)	0.857
Unknown	42 (2.8)	53 (3.3)	
**Body mass index** (kg/m^2^)			
<23.0	819 (52.8)	871 (54.3)	
23~24.9	302 (19.5)	308 (19.2)	
≥25	374 (24.1)	358 (22.3)	0.490
Unknown	56 (3.6)	68 (4.2)	
**Age at menarche** (years)			
≤12.0	194 (12.5)	233 (14.5)	
>12.0	1302 (83.9)	1327 (82.7)	0.117
Unknown	55 (3.6)	45 (2.8)	
**Menopausal status**			
Premenopausal	928 (59.8)	864 (53.8)	
Postmenopausal	598 (38.6)	710 (44.2)	<0.01
Unknown	25 (1.6)	31 (2.0)	
**Age at menopause** (years) ^c^			
≤45	112 (18.7)	122 (17.2)	
46~50	235 (39.3)	310 (43.7)	
≥51	209 (35.0)	248 (34.9)	0.438
Unknown	42 (7.0)	30 (4.2)	
**Parity**			
0	125 (8.1)	117 (7.3)	
≥1	1391 (89.6)	1454 (90.6)	0.410
Unknown	35 (2.3)	34 (2.1)	
**Breastfeeding**			
Yes	1166 (75.2)	1236 (77.0)	
No	236 (15.2)	285 (17.8)	0.179
Unknown	149 (9.6)	84 (5.2)	
**Physical activity** (MET-h/week/year)			
<3	858 (55.3)	659 (41.1)	
≥3	585 (37.7)	837 (52.1)	<0.01
Unknown	108 (7.0)	109 (6.8)	
**Family history of breast cancer**			
Absent	1451 (93.5)	1509 (94.0)	
Present	52 (3.4)	47 (2.9)	0.493
Unknown	48 (3.1)	49 (3.1)	

^a^ *P* for Chi-square test between case and control groups

^b^ *P* for Student’s t-test between case and control groups

^c^ Postmenopausal women only

In total, information on history of diabetes was obtained in 1461 (94.2%) cases and 1508 (94.0%) controls. After adjustment for potential breast cancer risk factors, diabetes mellitus was significantly associated with an elevated risk of breast cancer, with OR (95%CI) of 1.67 (1.11, 2.52) (Table 2). This positive association was significant in postmenopausal women [OR (95%CI): 1.63 (1.04, 2.56)] but not among premenopausal women [1.63 (0.47, 5.70)] (S2 Table). Genotypes of *FTO* rs3751812, *IL-6* rs1800796 and *HSPD1* rs2605039 were equally distributed in the cases and controls and consequently not significantly associated with breast cancer risk [ORs and 95%CIs of dominant effects: 0.91 (0.75, 1.10), 1.07 (0.91, 1.26) and 0.96 (0.80, 1.14), respectively] (Table 2), and these findings did not change materially with menopausal status (S2 Table). We also found that both diabetes and genetic variations of *FTO*, *IL-6*, *HSPD1* genes were equally distributed across clinical characteristics of breast cancer (S3 Table).

**Table 2:** Multivariate odds ratio of breast cancer risk associated with genetic variations of *FTO*, *IL-6*, *HSPD1* genes and diabetes.

Variable	Cases n* (%)	Controls n* (%)	OR(95%CI) ^a^	OR(95%CI) ^b^
***FTO* rs3751812**				
GG	1195 (78.7)	1186 (77.0)	1.00 (reference)	1.00 (reference)
GT	299 (19.7)	337 (21.9)	0.88 (0.74,1.05)	0.89 (0.73,1.08)
TT	25 (1.6)	18 (1.2)	1.38 (0.75,2.54)	1.24 (0.60,2.57)
*P* for trend			0.457	0.440
GT/TT	324 (21.3)	355 (23.0)	0.91 (0.76,1.07)	0.91 (0.75,1.10)
***IL-6* rs1800796**				
CC	898 (59.3)	963 (62.5)	1.00 (reference)	1.00 (reference)
CG	522 (34.5)	499 (32.4)	1.12 (0.96,1.31)	1.06 (0.89,1.26)
GG	94 (6.2)	78 (5.1)	1.29 (0.95,1.77)	1.11 (0.79,1.57)
*P* for trend			0.044	0.409
CG/GG	616 (40.7)	577 (37.5)	1.15 (0.99,1.32)	1.07 (0.91,1.26)
***HSPD1* rs2605039**				
GG	429 (28.4)	429 (28.0)	1.00 (reference)	1.00 (reference)
GT	756 (50.2)	760 (49.6)	0.99 (0.84,1.18)	0.98 (0.82,1.18)
TT	323 (21.4)	343 (22.4)	0.94 (0.77,1.15)	0.89 (0.71,1.12)
*P* for trend			0.574	0.355
GT/TT	1079 (71.6)	1103 (72.0)	0.98 (0.84,1.15)	0.96 (0.80,1.14)
**Diabetes status**				
Non-diabetic	1388 (95.0)	1458 (96.7)	1.00 (reference)	1.00 (reference)
Diabetic	73 (5.0)	50 (3.3)	**1.68 (1.15,2.45)**	**1.67 (1.11,2.52)**

* The number may not equal to the total number due to missing data

^a^ Adjusted for age (continuous)

^b^ Adjusted for age (continuous), age at menarche (≤12.0 vs >12.0), marital status (Never married vs married/living as married and separated/widow), education (Junior middle school or below vs senior middle school and college or above), BMI (<22.0 vs 22~24.9 and ≥25), parity (0 vs ≥1), menopausal status (Premenopausal vs postmenopausal), breastfeeding (Yes vs no), physical activity (<3 vs ≥3), and family history of breast cancer (Absent vs present).

We further analyzed the joint effects of *FTO*, *IL-6*, *HSPD1* polymorphisms with diabetes on breast cancer risk. Compared to non-diabetic women, diabetic women had an increased risk of breast cancer among the women with the GG genotype of *FTO* rs3751812 [OR (95%CI): 2.05 (1.26, 3.34)] but not among those with GT/TT genotypes [0.90 (0.38, 2.12)], but the interaction was not significant (*P* > 0.05) (Table 3). Compared to the women without diabetes, the increased risk of breast cancer by diabetes was more obvious among women with the CC genotype [OR (95%CI): 2.53 (1.45, 4.41)] than those with CG/GG genotypes [1.10 (0.57, 2.10)] of *IL-6* rs1800796 and the interaction was significant in a multiplicative model but not in an additive model (*P* values: 0.048 and 0.294, respectively) (Table 3). For *HSPD1* rs2605039, diabetes significantly increased the risk of breast cancer among women with the GG genotype [OR (95%CI): 6.40 (2.29, 17.87)] but not the women with the GT/TT genotypes [1.16 (0.71, 1.89)], and the interaction was significant in a multiplicative model but not in an additive model (*P* values: 0.002 and 0.134, respectively) (Table 3).

**Table 3:** Joint effects between genetic variations of *FTO*, *IL-6*, *HSPD1* genes and diabetes on breast cancer risk.

Genotype	Diabetes status	Cases n* (%)	Controls n* (%)	OR (95%CI) ^a^	OR (95%CI) ^b^
***FTO* rs3751812**					
GG	Non-diabetic	1064 (94.8)	1082 (97.2)	1.00 (reference)	1.00 (reference)
	Diabetic	58 (5.2)	31 (2.8)	**2.06 (1.30,3.24)**	**2.05 (1.26,3.34)**
GT/TT	Non-diabetic	296 (95.5)	319 (94.9)	1.00 (reference)	1.00 (reference)
	Diabetic	14 (4.5)	17 (5.1)	1.01 (0.47,2.16)	0.90 (0.38,2.12)
*P* for interaction (multiplicative/additive)				0.085/0.726	0.115/0.757
***IL-6* rs1800796**					
CC	Non-diabetic	799 (94.7)	879 (97.5)	1.00 (reference)	1.00 (reference)
	Diabetic	45 (5.3)	23 (2.5)	**2.39 (1.41,4.02)**	**2.53 (1.45,4.41)**
CG/GG	Non-diabetic	555 (95.4)	521 (95.6)	1.00 (reference)	1.00 (reference)
	Diabetic	27 (4.6)	24 (4.4)	1.13 (0.63,2.03)	1.10 (0.57,2.10)
*P* for interaction (multiplicative/additive)				0.071/0.208	**0.046**/0.294
***HSPD1* rs2605039**					
GG	Non-diabetic	379 (94.0)	398 (98.8)	1.00 (reference)	1.00 (reference)
	Diabetic	24 (6.0)	5 (1.2)	**5.62 (2.10,15.09)**	**6.40 (2.29,17.87)**
GT/TT	Non-diabetic	972 (95.5)	994 (95.9)	1.00 (reference)	1.00 (reference)
	Diabetic	46 (4.5)	43 (4.1)	1.19 (0.77,1.85)	1.16 (0.71,1.89)
*P* for interaction (multiplicative/additive)				**0.005/0.024**	**0.002**/0.134

* The number may not equal to the total number due to missing data

^a^ Adjusted for age (continuous)

^b^ Adjusted for age (continuous), age at menarche (≤12.0 vs >12.0), marital status (Never married vs married/living as married and separated/widow), education (Junior middle school or below vs senior middle school and college or above), BMI (<22.0 vs 22~24.9 and ≥25), parity (0 vs ≥1), menopausal status (Premenopausal vs postmenopausal), breastfeeding (Yes vs no), physical activity (<3 vs ≥3), and family history of breast cancer (Absent vs present).

**Fig 1:**
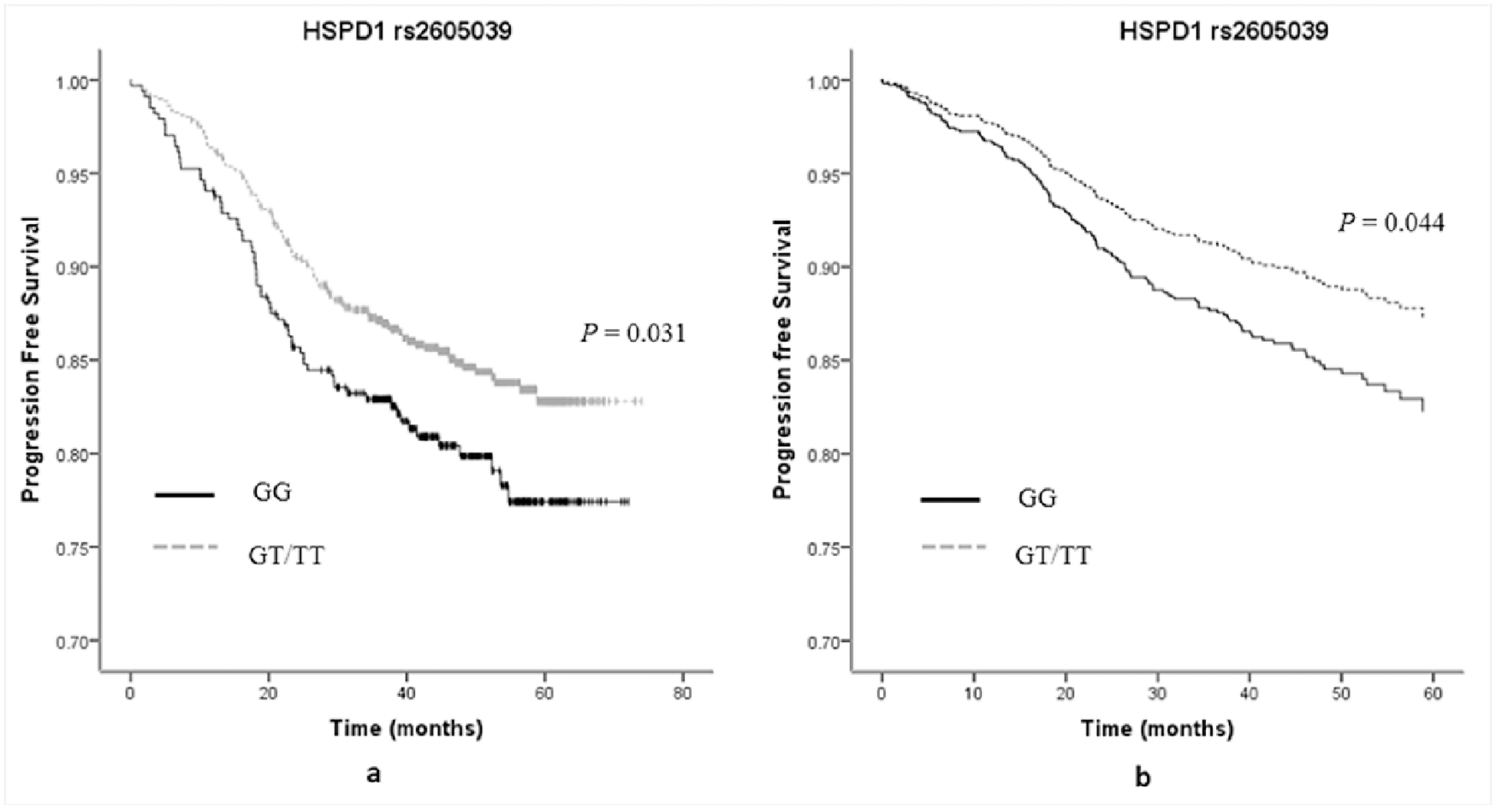
(A) Kaplan—Meier estimates on progression free survival for breast cancer patients according to genotypes of HSPD1 rs2605039. (B) Multivariate Cox regression analysis on progression free survival for breast cancer patients according to genotypes of HSPD1 rs2605039 (Adjusted for age at diagnosis, ER status, HER2 status, clinical stage, chemotherapy and surgical options).

**Fig 2:**
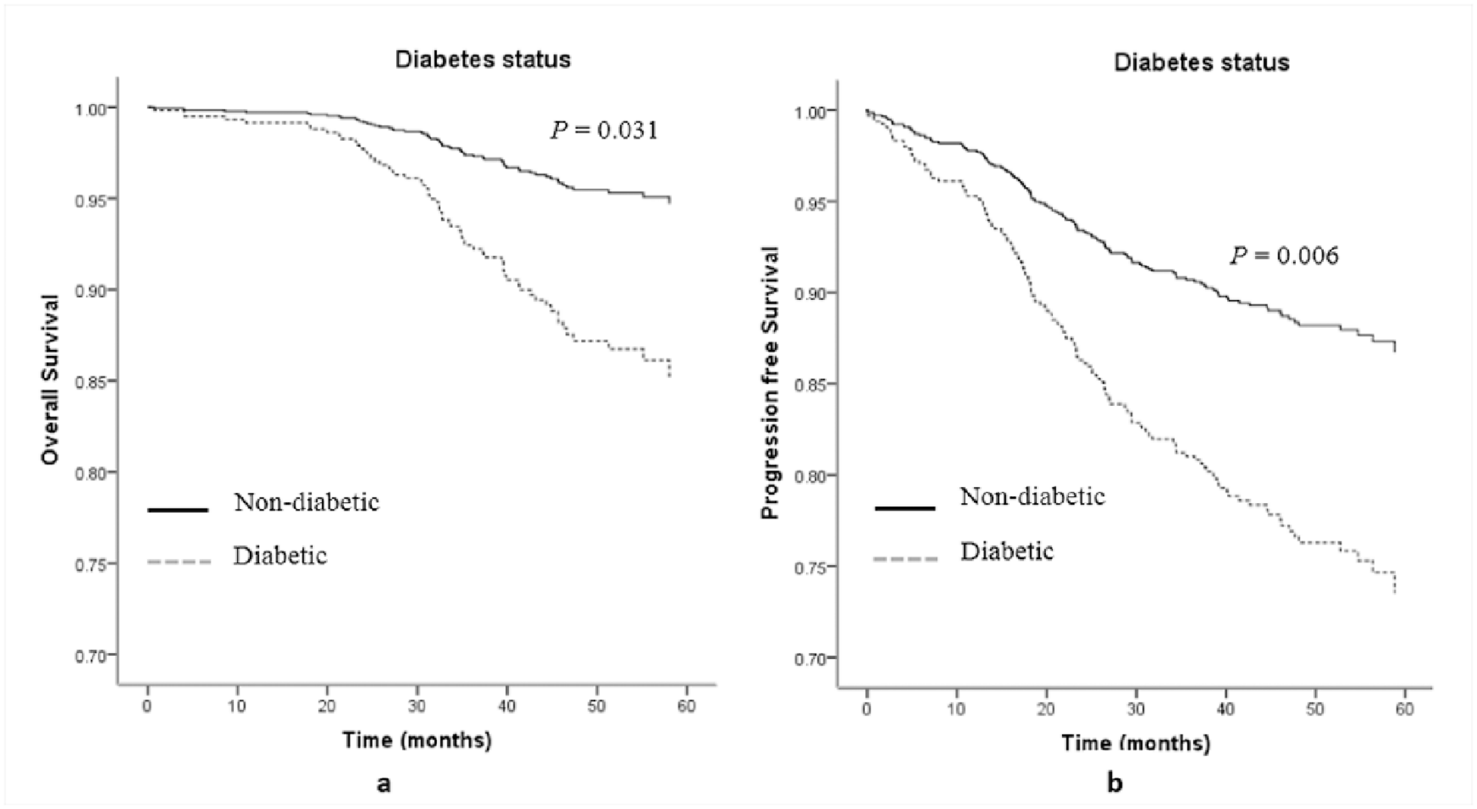
(A) Multivariate Cox regression analysis on overall survival for breast cancer patients according to diabetes status (Adjusted for age at diagnosis, ER status, HER2 status, clinical stage, chemotherapy and surgical options). (B) Multivariate Cox regression analysis on progression free survival for breast cancer patients according to diabetes status (Adjusted for age at diagnosis, ER status, HER2 status, clinical stage, chemotherapy and surgical options).

Finally, we conducted survival analyses. In total, 1113 of 1168 follow-up patients were available for diabetes information, with 54 diabetic patients and 1059 non-diabetic patients. In the univariate model, diabetes, *FTO* rs3751812, and *IL-6* rs1800796 were associated with neither OS nor PFS of breast cancer patients (all *P* values > 0.05) (S1 and S2 Figs), while *HSPD1* rs2605039 was significantly associated with PFS (*P* = 0.031) (Fig 1A) but not OS (*P* = 0.061). Except for age at diagnosis, other clinical factors (ER and HER2 statuses, clinical stages, chemotherapy, and surgical options) were all significantly associated with OS and PFS in a univariate Cox regression model (S4 Table). After adjusting for these clinical factors, diabetes was significantly associated with increased risks of death and progression of breast cancer [HRs and 95%CIs: 2.66 (1.10, 6.44) and 2.46 (1.29, 4.70), respectively] (Table 4, Fig 2). Patients with GT/TT genotypes of *HSPD1* rs2605039 had a better PFS but not OS than those with GG genotype with HR (95%CI) of 0.70 (0.49 .99) (Table 4, Fig 1B). *FTO* rs3751812 and *IL-6* rs1800796 did not change either OS or PFS of breast cancer (Table 4). Due to a limited proportion of patients with diabetes, we did not conduct stratified analyses to explore the association of diabetes with breast cancer survival according to genotypes of three genetic variations.

**Table 4:** Multivariate survival analysis of breast cancer associated with genetic variations of *FTO*, *IL-6*, *HSPD1* genes and diabetes.

Variable	Overall survival (OS)		Progression free survival (PFS)	
	Death (n, %)	HR (95%CI) ^a^	Progression (n, %)	HR (95%CI) ^a^
***FTO* rs3751812**				
GG	79 (8.9)	1.00 (reference)	145 (16.3)	1.00 (reference)
GT/TT	26 (10.3)	1.04 (0.59,1.82)	44 (17.5)	0.91 (0.61,1.35)
***IL-6* rs1800796**				
CC	55 (8.3)	1.00 (reference)	103 (15.5)	1.00 (reference)
CG/GG	51 (10.6)	1.29 (0.80,2.07)	84 (17.5)	1.11 (0.79,1.55)
***HSPD1* rs2605039**				
GG	39 (11.6)	1.00 (reference)	67 (19.9)	1.00 (reference)
GT/TT	65 (8.1)	0.75 (0.46,1.25)	120 (15.0)	**0.70 (0.49,0.99)**
**Diabetes status**				
Non-diabetic	90 (8.5)	1.00 (reference)	167 (15.8)	1.00 (reference)
Diabetic	6 (11.1)	**2.66 (1.10,6.44)**	11 (20.4)	**2.46 (1.29,4.70)**

^a^ Adjusted for age at diagnosis (continuous), ER status (Negative vs positive), HER2 status (Negative vs equivocal/positive), clinical stage (I/II vs III/IV), chemotherapy (Yes vs No), and surgical options (Modified radical mastectomy vs other options).

## Discussion

In the present study, we confirmed the positive association of diabetes with breast cancer risk and prognosis. We further found that the risk association might be modified by polymorphisms of *IL-6* rs1800796 and *HSPD1* rs2605039. Diabetes and *HSPD1* rs2605039 might also influence breast cancer survival.

We found that the increased risk of breast cancer by diabetes was more obvious among women with the CC genotype than those with CG/GG genotypes of *IL-6* rs1800796. The G and C alleles were reported to be associated with low and high serum levels of IL-6, respectively [23]. For breast cancer, IL-6 seems to be a double-edged sword because it was identified as both a tumor-promoting and a tumor-inhibiting cytokine [27,28]. Furthermore, in a diabetic state, high levels of IL-6 may promote carcinogenesis by an inflammatory pathway [14,29]. These evidences would help in interpreting the interaction between diabetes and *IL-6* rs1800796 shown in the present study. A low level of IL-6 in carriers with G allele, on the one hand, may exert antitumorigenic effects predominately in a diabetic state, resulting in that diabetic women with CG/GG genotypes had a marginally decreased risk of breast cancer compared to women with CC genotype in our study [OR (95%CI): 0.36 (0.12, 1.07)]. On the other hand, a high level of IL-6 by C allele may be dominant in tumor-promoting effects, deteriorate the chronic inflammatory state in diabetes, and synergically increase breast cancer risk, implying that the contribution to breast cancer by diabetes may be varied by different IL-6 levels.

Hsp60 was supposed to be positively correlated with tumor growth and proliferation [30]. In the present study, we found that breast cancer patients with GT/TT genotypes of *HSPD1* rs2605039 had a better PFS than those with GG genotype, indicating that the T and G alleles are likely to be associated with low and high levels of hsp60, respectively. It has been found that up-regulation (high level) of hsp60 resulted in anti-apoptosis and promoting carcinogenesis [31,32]. It has also been reported that inflammatory stress may induce the release of hsp60, which exerts autocrine/paracrine effects on adipocytes accompanying by an increased release of pro-inflammatory adipokines, promoting inflammatory signaling, and insulin resistance [33]. Similar to a previous study [21], the co-existence of diabetes and the G allele of *HSPD1* rs2605039 might increase the hsp60 level high enough to make the host’s anti-apoptotic effect, inflammatory and insulin resistant states more apparent while the co-existence of diabetes and the T allele might keep hsp60 at a certain low level to exert a pro-apoptosis effect. However, the exact mechanisms in which diabetes and hsp60 interact on breast cancer risk remain to be studied. Nevertheless, the finding suggested that *HSPD1* may be a potential target gene to be interfered for a better prognosis of breast cancer, particularly for the breast cancer patients with diabetes.

The above modification effects in the present study may provide a possible explanation for the previous findings that the association between diabetes and breast cancer risk was more obvious in European populations than in Asians [2,7–9]. We then compared the differences of the allele frequencies of the SNPs between Caucasians and Chinese through HapMap database, and examined whether the frequencies of the alleles, with which the diabetic subjects had an increased risk of breast cancer, were higher in European populations than in Asians. However, it turned out not to be the case. Therefore, we can’t conclude that *IL-6* rs1800796 and *HSPD1* rs2605039 is a reason for the race difference of the association between diabetes and breast cancer risk, although the possibility can’t be excluded as it is only an ecological relationship between allele frequencies and the association strength. It would be interesting that a similar study could be conducted among Caucasians: A same result would suggest a general phenomenon; a different result may be explained by the interaction between genes and environments [34]. It is also likely that menopausal status may be able to explain the phenomena to some extent, because European incident breast cancer patients are usually older (postmenopausal) than Chinese [35], while the association particularly occurred among postmenopausal women as found in previous studies [36,37] as well as in the present study.

Although we did not observe a significant association between diabetes and prognosis of breast cancer in a univariate Kaplan—Meier estimate (S1 and S2C Figs), it turned to be marked in a multivariate Cox regression model (Fig 2). This discrepancy was likely because the contribution of diabetes was covered up by clinical factors in univariate survival analysis, which was shown that the proportion of diabetes patients receiving chemotherapy was far less than those without diabetes (75.9% vs 89.1%, *P* = 0.003), whereas chemotherapy was associated with a poor prognosis in the present study (S4 Table). Chemotherapy played a negative confounding role and the diabetic status turned to be significantly associated with a worse prognosis of breast cancer when the confounding role of chemotherapy was controlled. This finding, also in line with the results from other studies [38–40], suggested that diabetic status is not only an endocrine disease but also lead to increased risk of other outcomes. Therefore, the need for well controlled diabetes is not a trivial matter.

For exploring the association between diabetes and breast cancer, anti-diabetic medications should be taken into consideration. Medications that acting through an increase of the circulating levels of insulin (including insulin and its analogues, insulin secretagogues) have been reported to be associated with an increased risk of cancer [41]. On the contrary, metformin, a glucose-lowering drug that inhibits hepatic gluconeogenesis, was associated with a reduced risk of cancer [42]. In addition, anti-diabetic agents that directly target insulin resistance, such as thiazolidinediones (TZDs), have also been proposed to lower the risk of cancer [43]. However, when breast cancer was investigated separately from other cancers, discordant results for the medications were observed [44], making it difficult to evaluate the impact of anti-diabetic medications on breast carcinogenesis. Chlebowski et al. conducted a stratified analysis to explore the association between diabetes and breast cancer according to metformin application, and they found that women treated with medications other than metformin showed a non-significant increased risk of breast cancer, while those receiving metformin presented a lower breast cancer incidence [45]. Therefore, anti-diabetic medications, trying to control glycaemia or target on insulin resistance, need to be verified by further exploration especially in high breast cancer risk people.

Several limitations should be considered when interpreting the results of the present study. First of all, the hospital-based design in our case-control study might lead to selection bias [20]. However, this selection bias was minimized, because the cases and controls were comparable to some extent as they were recruited from the same hospital during the same period, and likely to resemble each other with regard to those selective factors that led to the hospital admission and use of the facilities [46]. Furthermore, the prevalence of previously diagnosed diabetes in the controls was 2.92%, close to that (2.2%) in previous study (at age 40 to 49 years old) in Guangzhou area and all over China (3.2%) [47,48], indicating the sample of our study was representative. Second, self-report of diabetes history was subjected to recall bias, which we expected not to be so important to our results, because previous studies showed that findings were unchanged when the diagnosis of diabetes was self-reported or confirmed with medical records [36]. Third, type 1 and type 2 diabetes were not distinguished in our study. Type 1 diabetes may not contribute to breast cancer [36], so the intensity of the association between diabetes and breast cancer may have been somewhat underestimated. Nevertheless, type 2 diabetes is the predominant form of diabetes in adults [49], the underestimated results could reflect the true association to some extent. Lastly, in our breast cancer patient’s follow-up, although we tried various ways to reduce missing rate, we still did not successfully follow up all the cases, which might lead to withdraw bias. Nevertheless, the demographic characteristics, common risk factors and clinical characteristics were equally distributed between follow-up patients and defaulters (data not shown), so the withdraw bias might exert very little influence on the results.

In summary, our results suggested that diabetes was associated with breast cancer risk in the Chinese population, which may be modified by genetic variations of *IL-6* rs1800796 and *HSPD1* rs2605039. Diabetes and *HSPD1* rs2605039 were also associated with breast cancer survival. Whether the genetic variations contribute to the different strengths of the association between diabetes and breast cancer risk, however, can’t be confirmed and remains to be further studied.

## Supporting information

S1 Table The primer sequences for three genetic variations. (DOC)

S2 Table Multivariate odds ratio of breast cancer risk associated with genetic variations of FTO, IL-6, HSPD1 genes and diabetes stratified by menopausal status (DOC)

S3 Table Correlations between diabetes, genetic variations of FTO, IL-6, HSPD1 genes and clinical characteristics among breast cancer patients. (DOC)

S4 Table Univariate Cox regression analysis for clinical variables. (DOC)

S1 Fig (A) Kaplan—Meier estimates on overall survival for breast cancer patients according to genotypes of FTO rs3751812. (B) Kaplan—Meier estimates on overall survival for breast cancer patients according to genotypes of IL-6 rs1800796. (C) Kaplan—Meier estimates on overall survival for breast cancer patients according to diabetes.
(DOC)

S2 Fig (A) Kaplan—Meier estimates on progression free survival for breast cancer patients according to genotypes of FTO rs3751812. (B) Kaplan—Meier estimates on progression free survival for breast cancer patients according to genotypes of IL-6 rs1800796. (C) Kaplan—Meier estimates on progression free survival for breast cancer patients according to diabetes.
(DOC)
