# Supplementary material for: Modification effects of genetic polymorphisms in FTO, IL-6, and HSPD1 on the associations of diabetes with breast cancer risk and survival
Source: PLoS One. 2017 Jun 7;12(6):e0178850. doi: 10.1371/journal.pone.0178850 (PMC5462388; doi:10.1371/journal.pone.0178850)
Supplement: S1 Table — (DOC) [file pone.0178850.s001.doc]

**S1 Table** The primer sequences for three genetic variations

| SNP | Primer | Sequence |
| --- | --- | --- |
| *FTO* rs3751812 | Amplification primer 1 | ACGTTGGATGAGACCTGAAAATAGGTGAGC |
|  | Amplification primer 2 | ACGTTGGATGCTTTTTCGCTGGTAGGATGC |
|  | Extension primer | GCCTCTCCCTGCCAACA |
| *IL-6* rs1800796 | Amplification primer 1 | ACGTTGGATGTCTTCTGTGTTCTGGCTCTC |
|  | Amplification primer 2 | ACGTTGGATGACGCCTTGAAGTAACTGCAC |
|  | Extension primer | GCAGTTCTACAACAGCC |
| *HSPD1* rs2605039 | Amplification primer 1 | ACGTTGGATGCCACCATCCATCTCCAAAAC |
|  | Amplification primer 2 | ACGTTGGATGTGGAGGAGAGAATGGGAAGC |
|  | Extension primer | GGGAATGGGAAGCTACTGT |
