# Supplementary material for: Modification effects of genetic polymorphisms in FTO, IL-6, and HSPD1 on the associations of diabetes with breast cancer risk and survival
Source: PLoS One. 2017 Jun 7;12(6):e0178850. doi: 10.1371/journal.pone.0178850 (PMC5462388; doi:10.1371/journal.pone.0178850)
Supplement: S2 Table — (DOC) [file pone.0178850.s002.doc]

**S2 Table Multivariate odds ratio of breast cancer risk associated with genetic variations of *FTO*, *IL-6*, *HSPD1* genes and diabetes status stratified by menopausal status**

| Variables | **Premenopausal** | | |  | **Postmenopausal** | | |  |
| --- | --- | --- | --- | --- | --- | --- | --- | --- |
|  | Cases  n* (%) | Controls  n* (%) | OR (95%CI) ^a^ |  | Cases  n* (%) | Controls  n* (%) | OR (95%CI) ^a^ |  |
| ***FTO* rs3751812** | |  |  |  |  |  |  |  |
| GG | 718 (78.8) | 645 (77.8) | 1.00 (reference) |  | 459 (78.7) | 518 (76.1) | 1.00 (reference) |  |
| GT | 180 (19.8) | 173 (20.9) | 0.92 (0.71,1.18) |  | 115 (19.7) | 157 (23.1) | 0.86 (0.63,1.18) |  |
| TT | 13 ( 1.4 ) | 11 ( 1.3 ) | 0.95 (0.40,2.29) |  | 9 ( 1.5 ) | 6 ( 0.9 ) | 1.86 (0.49,7.08) |  |
| GT/TT | 193 (21.2) | 184 (22.2) | 0.92 (0.72,1.18) |  | 124 (21.3) | 163 (23.9) | 0.89 (0.65,1.21) |  |
| *P* for interaction (multiplicative/additive) | | | |  |  |  | 0.833/0.928 |  |
| ***IL-6* rs1800796** | |  |  |  |  |  |  |  |
| CC | 546 (60.1) | 510 (61.4) | 1.00 (reference) |  | 336 (57.8) | 430 (63.3) | 1.00 (reference) |  |
| CG | 314 (34.6) | 274 (33.0) | 1.04 (0.84,1.30) |  | 203 (34.9) | 217 (32.0) | 1.03 (0.78,1.35) |  |
| GG | 48 ( 5.3 ) | 46 ( 5.5 ) | 0.96 (0.62,1.50) |  | 42 ( 7.2 ) | 32 ( 4.7 ) | 1.46 (0.83,2.57) |  |
| CG/GG | 362 (39.9) | 320 (38.6) | 1.03 (0.84,1.27) |  | 245 (42.2) | 249 (36.7) | 1.08 (0.83,1.40) |  |
| *P* for interaction (multiplicative/additive) | | | |  |  |  | 0.688/0.870 |  |
| ***HSPD1* rs2605039** | |  |  |  |  |  |  |  |
| GG | 249 (27.4) | 228 (27.7) | 1.00 (reference) |  | 170 (29.6) | 194 (28.6) | 1.00 (reference) |  |
| GT | 468 (51.5) | 403 (49.0) | 1.06 (0.84,1.36) |  | 280 (48.8) | 342 (50.4) | 0.87 (0.65,1.17) |  |
| TT | 192 (21.1) | 192 (23.3) | 0.92 (0.69,1.23) |  | 124 (21.6) | 143 (21.1) | 0.89 (0.62,1.27) |  |
| GT/TT | 660 (72.6) | 595 (72.3) | 1.02 (0.81,1.28) |  | 404 (70.4) | 485 (71.4) | 0.88 (0.66,1.16) |  |
| *P* for interaction (multiplicative/additive) | | | |  |  |  | 0.378/0.560 |  |
| **Diabetes status** | |  |  |  |  |  |  |  |
| Non-diabetic | 876 (99.0) | 798 (99.4) | 1.00 (reference) |  | 505 (88.8) | 659 (93.6) | 1.00 (reference) |  |
| Diabetic | 9 (1.0 ) | 5 ( 0.6 ) | 1.63 (0.47,5.70) |  | 64 (11.2) | 45 ( 6.4 ) | **1.63 (1.04,2.56)** |  |
| *P* for interaction (multiplicative/additive) | | | |  |  |  | 0.829/0.578 |  |

* The number may not equal to the total number due to missing data

^a^ Adjusted for age (continuous), age at menarche (≤12.0 vs ＞12.0), marital status (Never married vs married/living as married and separated/widow), education (Junior middle school or below vs senior middle school and college or above), BMI (＜22.0 vs 22~24.9 and ≥25), parity (0 vs ≥1), breastfeeding (Yes vs no), physical activity (＜3 vs ≥3), and family history of breast cancer (Absent vs present)
