# Supplementary material for: Modification effects of genetic polymorphisms in FTO, IL-6, and HSPD1 on the associations of diabetes with breast cancer risk and survival
Source: PLoS One. 2017 Jun 7;12(6):e0178850. doi: 10.1371/journal.pone.0178850 (PMC5462388; doi:10.1371/journal.pone.0178850)
Supplement: S3 Table — (DOC) [file pone.0178850.s003.doc]

**S3 Table** Correlations between diabetes, genetic variations of *FTO*, *IL-6*, *HSPD1* genes and clinical characteristics among breast cancer patients

| characteristics | Diabetes status, n* (%) | |  | *FTO* rs3751812, n* (%) | |  | *IL-6* rs1800796, n* (%) | |  | *HSPD1* rs2605039, n* (%) | |
| --- | --- | --- | --- | --- | --- | --- | --- | --- | --- | --- | --- |
|  | Diabetic | Non-diabetic |  | GG | GT/TT |  | CC | CG/GG |  | GG | GT/TT |
| ER |  |  |  |  |  |  |  |  |  |  |  |
| Positive | 54 (77.1) | 948 (71.8) |  | 808 (71.1) | 225 (73.3) |  | 605 (71.1) | 426 (72.6) |  | 280 (69.7) | 748 (72.6) |
| Negative | 16 (22.9) | 373 (28.2) |  | 329 (28.9) | 82 (26.7) |  | 246 (28.9) | 161 (27.4) |  | 122 (30.3) | 283 (27.4) |
| *P^#^* |  | 0.328 |  |  | 0.443 |  |  | 0.540 |  |  | 0.274 |
| PR |  |  |  |  |  |  |  |  |  |  |  |
| Positive | 45 (64.3) | 860 (65.2) |  | 741 (65.3) | 196 (63.8) |  | 545 (64.2) | 389 (66.3) |  | 257 (64.1) | 676 (65.6) |
| Negative | 25 (35.7) | 459 (34.8) |  | 394 (34.7) | 111 (36.2) |  | 304 (35.8) | 198 (33.7) |  | 144 (35.9) | 354 (34.4) |
| *P^#^* |  | 0.876 |  |  | 0.638 |  |  | 0.417 |  |  | 0.583 |
| HER2 |  |  |  |  |  |  |  |  |  |  |  |
| Positive/ equivocal | 19 (27.5) | 410 (31.4) |  | 354 (31.5) | 96 (31.8) |  | 268 (32.0) | 176 (30.2) |  | 119 (30.1) | 324 (31.8) |
| Negative | 50 (72.5) | 895 (68.6) |  | 770 (68.5) | 206 (68.2) |  | 570 (68.0) | 407 (69.8) |  | 277 (69.9) | 696 (68.2) |
| *P^#^* |  | 0.498 |  |  | 0.922 |  |  | 0.473 |  |  | 0.532 |
| Tumor size (cm) |  |  |  |  |  |  |  |  |  |  |  |
| ≤ 2 | 30 (44.8) | 465 (37.1) |  | 396 (36.9) | 116 (39.3) |  | 302 (37.2) | 207 (37.6) |  | 145 (38.3) | 362 (37.1) |
| > 2 | 37 (55.2) | 789 (62.9) |  | 676 (63.1) | 179 (60.7) |  | 509 (62.8) | 344 (62.4) |  | 234 (61.7) | 614 (62.9) |
| *P^#^* |  | 0.205 |  |  | 0.454 |  |  | 0.902 |  |  | 0.690 |
| Metastasis status |  |  |  |  |  |  |  |  |  |  |  |
| Localized | 44 (64.7) | 743 (56.8) |  | 635 (56.5) | 176 (58.5) |  | 487 (58.0) | 326 (56.0) |  | 238 (60.1) | 568 (55.9) |
| Regional/distant | 24 (35.3) | 564 (43.2) |  | 489 (43.5) | 125 (41.5) |  | 352 (42.0) | 256 (44.0) |  | 158 (39.9) | 449 (44.1) |
| *P^#^* |  | 0.202 |  |  | 0.538 |  |  | 0.447 |  |  | 0.147 |
| Clinical stage |  |  |  |  |  |  |  |  |  |  |  |
| I/II | 56 (82.4) | 990 (79.0) |  | 850 (79.0) | 233 (80.6) |  | 652 (80.98) | 427 (76.9) |  | 311 (82.1) | 762 (78.2) |
| III/IV | 12 (17.6) | 263 (21.0) |  | 226 (21.0) | 56 (19.4) |  | 154 (19.1) | 128 (23.1) |  | 68 (17.9) | 212 (21.8) |
| *P^#^* |  | 0.508 |  |  | 0.544 |  |  | 0.077 |  |  | 0.119 |

* The number may not equal to the total number due to missing data

^#^ *P* values for chi-square tests in the subgroups
