# Supplementary material for: Modification effects of genetic polymorphisms in FTO, IL-6, and HSPD1 on the associations of diabetes with breast cancer risk and survival
Source: PLoS One. 2017 Jun 7;12(6):e0178850. doi: 10.1371/journal.pone.0178850 (PMC5462388; doi:10.1371/journal.pone.0178850)
Supplement: S4 Table — (DOC) [file pone.0178850.s004.doc]

**S4 Table** Univariate Cox regression analysis for clinical variables

| Variable | Overall survival (OS) | |  | Progression free survival (PFS) | |
| --- | --- | --- | --- | --- | --- |
|  | Death (n, %) | HR (95%CI) ^a^ |  | Progression (n, %) | HR (95%CI) ^a^ |
| Age at diagnosis (continuous) | | 1.00 (0.98,1.02) |  |  | 0.99 (0.98,1.01) |
| ER status | |  |  |  |  |
| Negative | 42 (13.3) | 1.00 (reference) |  | 68 (21.5) | 1.00 (reference) |
| Positive | 57 ( 7.1 ) | 0.52 (0.35,0.78) |  | 112 (14.0) | 0.61 (0.45,0.83) |
| HER2 status | |  |  |  |  |
| Negative | 62 ( 8.1 ) | 1.00 (reference) |  | 111 (14.5) | 1.00 (reference) |
| Equivocal/positive | 37 (10.7) | 1.42 (0.95,2.14) |  | 69 (20.0) | 1.48 (1.09,2.00) |
| Clinical stage | |  |  |  |  |
| I/II | 37 ( 4.3 ) | 1.00 (reference) |  | 83 ( 9.7 ) | 1.00 (reference) |
| III/IV | 55 (25.0) | 6.84 (4.50,10.38) |  | 88 (40.0) | 5.36 (3.97,7.24) |
| Chemotherapy | |  |  |  |  |
| Yes | 99 (9.9) | 1.00 (reference) |  | 179 (17.9) | 1.00 (reference) |
| No | 1 (0.8) | 0.07 (0.01,0.52) |  | 5 ( 3.8 ) | 0.20 (0.08,0.48) |
| Surgical options |  |  |  |  |  |
| Other options | 15 ( 3.8 ) | 1.00 (reference) |  | 40 (10.2) | 1.00 (reference) |
| Modified radical mastectomy | 77 (10.8) | 2.83 (1.63,4.93) |  | 133 (18.7) | 1.90 (1.33,2.70) |

^a^ Unadjusted
