# Supplementary material for: Modification effects of genetic polymorphisms in FTO, IL-6, and HSPD1 on the associations of diabetes with breast cancer risk and survival
Source: PLoS One. 2017 Jun 7;12(6):e0178850. doi: 10.1371/journal.pone.0178850 (PMC5462388; doi:10.1371/journal.pone.0178850)
Supplement: S1 Fig — (A) Kaplan—Meier estimates on overall survival for breast cancer patients according to genotypes of FTO rs3751812. (B) Kaplan—Meier estimates on overall survival for breast cancer patients according to genotypes of IL-6 rs1800796. (C) Kaplan—Meier estimates on overall survival for breast cancer patients according to diabetes. (DOC) [file pone.0178850.s005.doc]

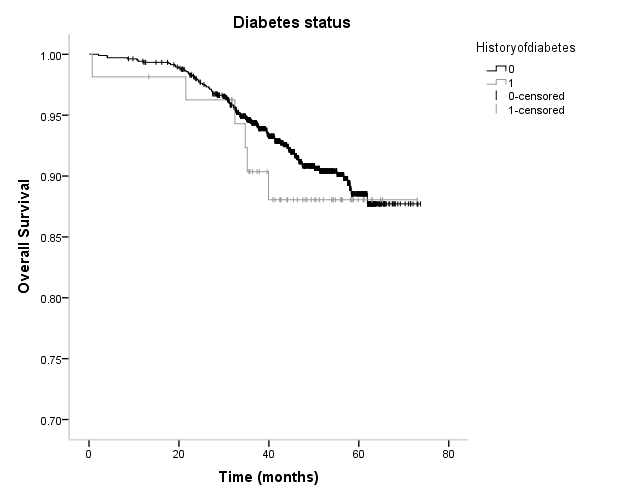

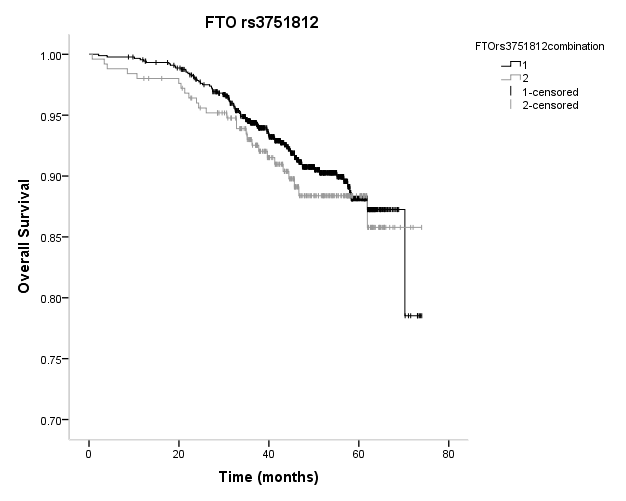

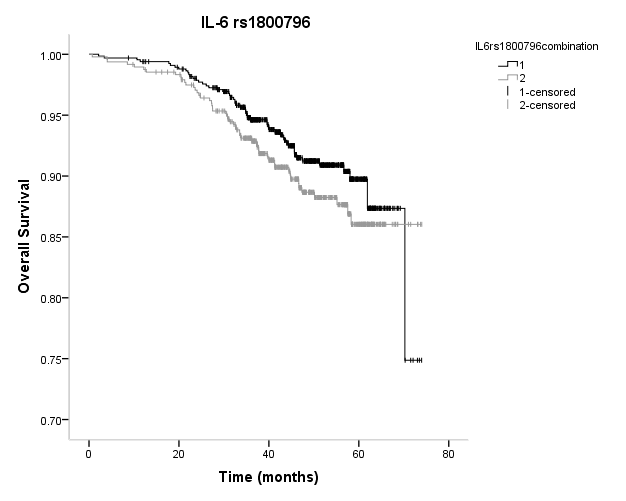


GG

GT/TT

CC

CG/GG

Non-diabetic

Diabetic

**a**

**b**

**c**

*P* = 0.470

*P* = 0.183

*P* = 0.556

**S1 Fig.** Kaplan–Meier estimates on overall survival for breast cancer patients according to genotypes of *FTO* rs3751812 (a), *IL-6* rs1800796 (b), and diabetes status (c).
