# Supplementary material for: Modification effects of genetic polymorphisms in FTO, IL-6, and HSPD1 on the associations of diabetes with breast cancer risk and survival
Source: PLoS One. 2017 Jun 7;12(6):e0178850. doi: 10.1371/journal.pone.0178850 (PMC5462388; doi:10.1371/journal.pone.0178850)
Supplement: S2 Fig — (A) Kaplan—Meier estimates on progression free survival for breast cancer patients according to genotypes of FTO rs3751812. (B) Kaplan—Meier estimates on progression free survival for breast cancer patients according to genotypes of IL-6 rs1800796. (C) Kaplan—Meier estimates on progression free survival for breast cancer patients according to diabetes. (DOC) [file pone.0178850.s006.doc]

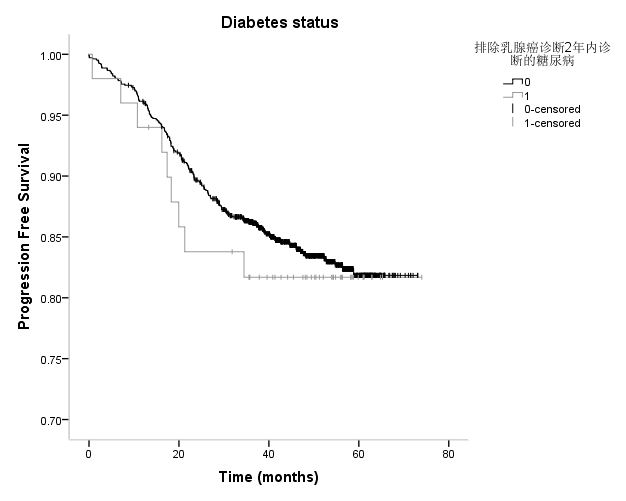

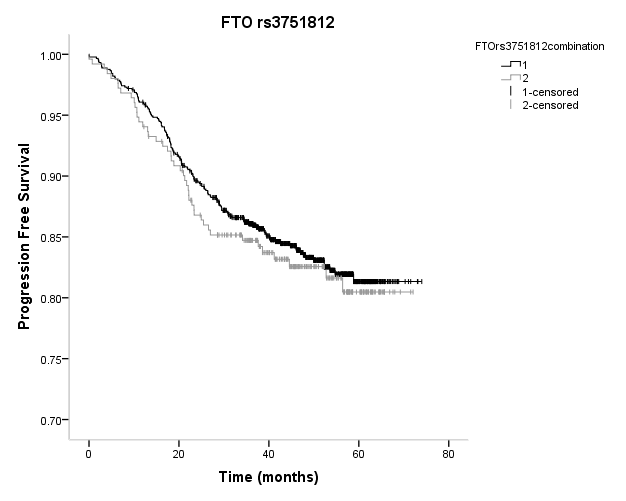

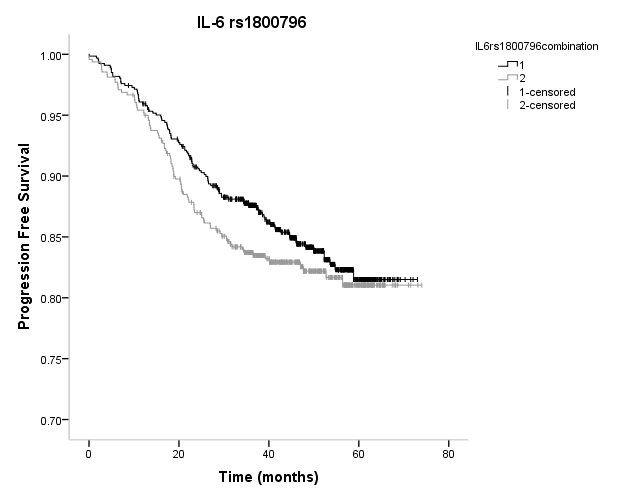


GG

GT/TT

CC

CG/GG

Non-diabetic

Diabetic

**a**

**b**

**c**

*P* = 0.617

*P* = 0.333

*P* = 0.379

**S2 Fig.** Kaplan–Meier estimates on progression free survival for breast cancer patients according to genotypes of *FTO* rs3751812 (a), *IL-6* rs1800796 (b), and diabetes status (c).
